# Heritability of Unilateral Elbow Dysplasia in the Dog: A Retrospective Study of Sire and Dam Influence

**DOI:** 10.3389/fvets.2019.00422

**Published:** 2019-11-22

**Authors:** Gabriela Baers, Greg G. Keller, Thomas R. Famula, Anita M. Oberbauer

**Affiliations:** ^1^Orthopedic Foundation for Animals, Columbia, MO, United States; ^2^Department of Animal Science, University of California, Davis, Davis, CA, United States

**Keywords:** elbow dysplasia, heritability, dog, sire effect, breeds

## Abstract

Canine elbow dysplasia is a significant health issue affecting many breeds. Unfortunately, treatments remain relatively limited, so control of this disease often falls to selectively breeding for dogs with normal elbows. The objectives of this study were to evaluate the heritability of left-sided vs. right-sided elbow dysplasia, and to assess potential differential sire and the dam influence on offspring elbow status. In a retrospective study, elbow data from 130,117 dogs over 2 years old representing 17 breeds were obtained from the database of the Orthopedic Foundation for Animals and included in the study. Heritability estimates for unilateral elbow dysplasia varied between breeds (ranging from 0.01 to 0.36) and were similar between the left and right elbows. The estimated genetic correlation between disease in the left and right elbow ~1 in the majority of breeds, with the exception of the hybrids, Australian Shepherds, and the Australian Cattle Dogs, likely due to low numbers of affected individuals. The sire and dam had equal impact on the offspring's elbow status. Furthermore, there was no increased risk for the sire or dam to pass on the same unilaterality of their elbow dysplasia to their offspring. However, the overall risk of elbow dysplasia in the offspring did increase when one or both parents were affected, though this also varied based on breed. Understanding of the impact that the sire and dam have on the offspring and of the overall heritability of both bilateral and unilateral elbow dysplasia is important in guiding breeding decisions to reduce the incidence in future generations of dogs.

## Introduction

A prevalent health issue that affects many breeds, particularly young medium to large sized dogs, is that of elbow dysplasia (1). Elbow dysplasia refers to the abnormal development of the elbow joint, resulting in early development of osteoarthritis and degenerative changes. Complex primary conditions associated with elbow dysplasia include fragmented medial coronoid (FCP), ununited anconeal process (UAP), osteochondrosis dessecans (OCD), and joint incongruency (1). The significance of having a dog diagnosed with elbow dysplasia is that treatments are not curative, and long-term prognosis is often poor. Surgery may be recommended for certain cases (2), but at this time the osteoarthritis and degenerative changes can only be treated with nutritional and medical management (3, 4), including maintaining a lean body weight, regular slow steady exercise as tolerated, physical rehabilitation, joint supplements, intra-articular injections, or oral medications to improve comfort (5).

Elbow dysplasia is an inherited disease, although it has been suggested that diet and exercise may influence the severity of the disease (1, 3, 6). Given that elbow dysplasia cannot be prevented in genetically predisposed dogs, and treatment is generally difficult, the primary method of controlling this disease is by attempting to eliminate elbow dysplasia through selective breeding. Several organizations throughout the world identify and record elbow dysplasia in dogs based upon radiographic evaluation and thereby provide tools to enable breeders to make informed decisions about which dogs to retain in their breeding programs. All organizations use screening protocols that comply with guidelines recommended by the International Elbow Working Group (IEWG) which grades elbows as normal (grade 0) or dysplastic, which then ranges from grade I to grade III dysplasia based on the severity of the degenerative changes. Importantly, general screening for elbow dysplasia only determines the phenotype of that particular individual dog, without predicting that dog's genetic makeup or its ability to produce unaffected puppies. Identifying the dog's phenotype significantly impacts the overall breeding value of that animal and improves the probability that the progeny also have improved phenotypes (7).

Environmental or physiological factors may affect the grading. For instance, a 1997 study by Corley et al., demonstrated that the Orthopedic Foundation for Animals (OFA) ratings on hips are increasingly more reliable as the dog reaches 2 years of age (8). Specifically, an assessment of normal hips for a dog between 13 and 18 months of age was 95% accurate when compared to the assessment of that dog at or over 2 years of age, leading to the determination that evaluations prior to 2 years of age are “preliminary” (8). Analogous data have not yet been studied in relation to the accuracy of diagnosing elbow dysplasia at early ages, but is suspected to be similar, especially given that previous data predicted only a slight influence of age on the presence of elbow dysplasia in dogs over 2 years of age (9). Some reports also indicate that male dogs are more frequently affected with elbow dysplasia than female dogs in certain breeds, such as in Labrador Retrievers (10). This is suspected to be correlated to hormonal differences between the sexes and a faster growth rate in male dogs.

Although the OFA does not recommend breeding dogs with any abnormal elbow result, regardless of dysplasia grade, some organizations such as the Federation Cynologique Internationale (FCI) and the British Veterinary Association (BVA) allow the breeding of dogs that are diagnosed with grade I elbow dysplasia as long as breeders proceed with caution and full awareness of the dog's other characteristics. However, previous research has shown that the risk of elbow dysplasia approximately doubles when one parent has elbow dysplasia, increasing from roughly 10–23% averaged across all dogs, with breed variations not considered (11). The risk of puppies developing elbow dysplasia has been shown to increase with severity of the disease in the parents. This indicates that there is a moderately high component of heritability to elbow dysplasia (11, 12). Overall, reports of heritability values for elbow dysplasia have been shown to vary significantly based on breed and population size (9, 13).

Within certain breeds, particularly those demonstrated to have a higher heritability, sires contribute slightly more in improving hip conformation within the population than dams do (9). This may, in part, be due to the popular sire effect in which certain males are bred widely to multiple females, but this sire impact has not been studied with consideration to individual contributions to the offspring or with consideration to elbow dysplasia. One small study involving Labrador Retrievers suggests there may be a maternal effect on the radiographic progression of elbow osteoarthritis in the offspring, but an underlying genetic component has not been evaluated (12) and the influence of the dam on elbow conformation includes factors beyond genetic contribution. The incidence of fragmented medial coronoid disease differs in prevalence between males and females, suggesting a sex-influenced component to inheritance (14). The presence of a strong maternal or paternal effect on offspring would impact breeder decisions on which dogs to continue using in breeding programs. Regardless of the genetic foundations of elbow disease, sire selection will always provide an outsized impact on breed improvement over that of dam selection, making the identification of superior sires all the more important.

It is also largely accepted that both hip and elbow dysplasia are most commonly bilateral, but that they may also present as unilateral disease. Anecdotally, many breeds appear to have a higher incidence of left-sided elbow dysplasia. Previous analyses using data published by the BVA (15) have shown that there is no difference in the heritability of right vs. left-sided hip dysplasia, but this has not been examined within elbow dysplasia. It is possible, therefore, that the heritability values may differ and may explain the anecdotal increase in prevalence of left-sided elbow dysplasia.

An objective of the present study was to evaluate whether differences existed between the heritability of dysplasia of the left and right elbows. An additional objective was to determine if the risk of elbow disease in offspring could be differentiated by the status of elbow disease in their sire vs. the elbow disease status in their dam. A final objective was to assess if a sire or dam with unilateral elbow dysplasia would pass on the same unilaterality of disease to their offspring, with the hypothesis that the same unilaterality would be inherited by the offspring with a higher frequency than the contralateral unilaterality. Characterizing the inheritance of elbow dysplasia will give breeders additional tools to reduce the incidence of this disease.

## Materials and Methods

Data collected from 1970 through November 2018 on 17 breeds of dogs older than 2 years of age were obtained retrospectively from the OFA database, and included dogs whose results were within the public domain and those whose results were withheld from public posting. If there were multiple submissions entered for a single dog, only the most recent submission was included for analysis. Only breeds having more than 380 elbow dysplasia submissions were used in the analyses.

Dogs were categorized as normal or dysplastic, and if dysplastic, they were sorted into bilateral disease, left-sided dysplasia, or right-sided dysplasia. There were no further classifications or distinctions made between the assigned grade of dysplasia or between any underlying pathology such as a fragmented medial coronoid, an OCD lesion, or an ununited anconeal process.

Data was then analyzed to evaluate the heritability of right-sided elbow dysplasia and left-sided elbow dysplasia and the relationship between them. Because it is feasible to treat the two elbow measures as two genetically distinct traits, the heritability of each elbow trait was estimated simultaneously as well as the genetic correlation between these measures. Anecdotally, investigators have suggested that when the genetic correlation between two traits exceeds 0.95 it is reasonable to consider the two traits as repeated measures of the same phenotype. The dataset permitted examination of that supposition.

Elbow dysplasia, on either lateral side, was measured as a binary characteristic (i.e., disease in the elbow is scored as 1, a normal elbow is scored as 0) and therefore, these two measures were approached as Bernoulli variables, considering the probability of disease in the *j*-th dog of the *i*-th sex as *p*_*ij*_. This probability is commonly transformed to its log-odds, or logit (θ_*ij*_ = *log*(*p*_*ij*_/(1 − *p*_*ij*_)), with the following representations for the left (L) and right (R) elbows:

$$\begin{array}{l} \theta_{Rij}=\mu_{R}+\beta_{R}age_{ij}{+\;sex}_{Ri\;}+\;a_{Rij}+e_{Rij}\\ \theta_{Lij}=\mu_{L}+\beta_{L}age_{ij}+sex_{Li\;}+\;a_{Lij}+e_{Lij} \end{array}$$

where μ_*R*_is an effect common to all dogs of a given breed, β_*R*_ is a regression coefficient accounting for the impact of age at screening to the risk of elbow disease, *sex*_*Ri*_is the contribution of the *i*-th sex (*i* = M, F) to the risk of elbow disease, *a*_*Rij*_ is the additive genetic contribution to elbow disease for the *j*-th dog of the *i*-th sex and *e*_*Rij*_ is an unknown residual impacting the risk to elbow disease for the *j*-th dog of the *i*-th sex. Naturally, the subscript “R” defines those terms impacting disease of the right elbow, and those with the subscript “L” identifying the concomitant terms for the left elbow. To provide for the potential genetic correlation between elbow traits, we assume

$$\begin{array}{l} \left[\begin{array}{c} a_{R}\\ a_{L}\; \end{array}\right]\sim N\left[\left[\begin{array}{c} 0\\ 0 \end{array}\right],\;[\begin{array}{cc} A\sigma_{gR}^{2} & A\sigma_{gRL}\;\\ A\sigma_{gRL} & A\sigma_{gL}^{2}\; \end{array}]\right] \end{array}$$

and

$$\begin{array}{l} \left[\begin{array}{l} e_{R}\\ e_{L} \end{array}\right]\sim N\left[\left[\begin{array}{l} 0\\ 0 \end{array}\right],\;[\begin{array}{ll} I & 0\\ 0 & I \end{array}]\right] \end{array}$$

where *a*_*R*_ (*a*_*L*_)is a vector of additive genetic (breeding) values associated with disease of the right (left) elbow of all the dogs represented in the database for a given breed, *A* is the numerator relationship matrix constructed from the list of sires and dams extracted for each breed, $\sigma_{gR}^{2}$ ($\sigma_{gL}^{2}$) is the additive genetic variance for the right (left) elbow trait, and σ_*gRL*_ is the additive genetic covariance for the right and left elbow traits. Finally *e*_*R*_ and *e*_*L*_ are vectors representing the unknown residual values for the right and left elbow traits, respectively, parameters that are set to have null means, unit variances and no covariance (16). Of course, it is then straightforward to extract the heritability of each lateral trait and their genetic correlation on this logit scale as

$$\begin{array}{l} h_{R}^{2}=\frac{\sigma_{gR}^{2}}{\sigma_{gR}^{2}+1}\\ \;h_{L}^{2}=\frac{\sigma_{gL}^{2}}{\sigma_{gL}^{2}+1} \end{array}$$

and

$$\begin{array}{l} r_{g}=\frac{\sigma_{gRL}}{\sqrt{\sigma_{gR}^{2}\sigma_{gL}^{2}}}\;. \end{array}$$

Software that can accommodate the evaluation of correlated binary traits when there are underlying relationships among the recorded animals is not widely available. Fortunately, the publicly available package MCMCglmm (17) is readily adapted to this challenge using the public domain statistical platform R (18). The framework of this R package is Bayesian analysis, an approach that fits naturally with the outline of the model above.

As part of the Bayesian framework, the prior distributions for the putative fixed effects (i.e., constants, sex, and age effects) were noted to have independent normal densities with null means and variances of 10^10^; that is, a diffuse normal prior. The prior distribution for the unknown covariance structure was assumed to be an inverse-Wishart density, provided by the package with parameters V, the scale matrix, and n, the degrees of freedom. Values for the genetic covariance matrix, which we set at the outset, were for V as an identity matrix (of order 2) and *n* = 3, a value which represents a flat prior across the interval [−1, 1] for the genetic correlation (17). The residual variance structure was fixed, as outlined in the model above, with the identity matrix of order 2 (16).

Estimates of the unknown parameters (i.e., sex effects, age effects, additive genetic values, variances, and covariances), and their transformation to heritabilities and the genetic correlation, are arrived at through a Markov Chain Monte Carlo (MCMC) process. After a series of preliminary analyses, we based our parameter estimates on a sample of 1,500 values from a single chain for each breed. The total number of samples generated was 200,000 with a “warm-up” period of 50,000 and a thinning rate of every 100-th sample [i.e., 1,500 = (200,000 – 50,000)/100]. The resulting chain was examined visually through trace and density plots for consistency, and autocorrelations were evaluated to insure that consecutive samples had a correlation <0.03 with the R package coda (19).

In addition to the estimation of these unknown genetic parameters, the number of matings between affected and unaffected males and females represented in this multi-breed database were counted. That is, all dogs were counted into one of four mutually exclusive categories: normal elbows, affected in left elbow only, affected in right elbow only and affected in both elbows. Similarly, when known, the sire and dam of each dog was counted into one of the same four mutually exclusive categories. With these counts, the frequency of each mating type along with the offspring outcomes from each mating type can be evaluated. Predictions of the probability for each of the four offspring phenotypic outcomes in each of the 16-possible mating types was facilitated with the *multinom* command, fitting a log-linear model to these nominal categories, available in the R package nnet (20).

## Results

A total of 130,117 dogs were included in the study, ranging from 24 to 190 months old. The mean age was 31.8 months old. Table 1 lists the breeds included along with their respective breed acronym codes, number of dogs evaluated, and prevalence of any elbow dysplasia. The breeds were then aggregated based upon number of dogs: the small population included breeds with fewer than 2,000 dogs (ACD, BMF, CC, EN, ES, and HY), a medium population included 2,000–10,000 dogs (AS, BF, MF, NF, RR, RO, and SMD), and a large population included more than 10,000 dogs (BMD, GR, GS, and LR).

**Table 1:** Breeds analyzed, total number of dogs submitted for evaluation, categorization of the population size, and prevalence of any recorded elbow dysplasia within these dogs, presented alphabetically by breed name.

**Breed**	**Breed code**	**Total number**	**Population size category**	**Elbow dysplasia prevalence**
Australian Cattle dog	ACD	685	Small	0.085
Australian Shepherd	AS	3,346	Medium	0.027
Bernese Mountain Dogs	BMD	10,178	Large	0.234
Bouvier Des Flanders	BF	2,057	Medium	0.089
Bullmastiff	BMF	1,214	Small	0.145
Chow Chow	CC	381	Small	0.472
English Setter	ES	1,887	Small	0.144
English Springer Spaniel	EN	1,048	Small	0.159
German Shepherd dog	GD	13,243	Large	0.165
Golden Retriever	GR	26,401	Large	0.087
Greater Swiss Mountain dog	SMD	2,328	Medium	0.088
Hybrid	HY	522	Small	0.019
Labrador Retriever	LR	46,514	Large	0.091
Mastiff	MF	3,982	Medium	0.128
Newfoundland	NF	4,146	Medium	0.214
Rhodesian Ridgeback	RR	5,005	Medium	0.050
Rottweiler	RO	7,180	Medium	0.345

The heritability of unilateral elbow dysplasia, varied substantially with each breed as shown in Table 2. The lowest heritabilities were noted in Australian Cattle Dogs, Australian Shepherds, and Hybrids. The highest heritabilities for unilateral elbow dysplasia were observed in Chow Chows, English Setters, and Rottweilers.

**Table 2:** Heritability estimates + standard deviation of the MCMC sample of left and right elbow dysplasia by breed, shown alphabetically by breed code.

**Breed**	**Heritability of elbow dysplasia in the left elbow**	**Heritability of elbow dysplasia in the right elbow**
ACD	0.08 + 4.6 × 10^−3^	0.06 + 5.7 × 10^−3^
AS	0.02 + 6.9 × 10^−4^	0.01 + 8.6 × 10^−4^
BF	0.06 + 1.8 × 10^−3^	0.11 + 1.1 × 10^−3^
BMD	0.21 + 5.0 × 10^−4^	0.22 + 4.0 × 10^−4^
BMF	0.08 + 2.6 × 10^−3^	0.11 + 2.6 × 10^−3^
CC	0.28 + 8.7 × 10^−3^	0.29 + 8.2 × 10^−3^
EN	0.28 + 3.1 × 10^−3^	0.19 + 3.4 × 10^−3^
ES	0.36 + 2.1 × 10^−3^	0.32 + 2.1 × 10^−3^
GR	0.16 + 1.8 × 10^−4^	0.13 + 1.8 × 10^−4^
GS	0.20 + 3.5 × 10^−4^	0.20 + 3.5 × 10^−4^
HY	0.01 + 1.8 × 10^−3^	0.01 + 8.7 × 10^−4^
LR	0.16 + 9.3 × 10^−5^	0.14 + 9.3 × 10^−5^
MF	0.10 + 7.9 × 10^−4^	0.10 + 9.5 × 10^−4^
NF	0.20 + 9.3 × 10^−4^	0.21 + 7.8 × 10^−4^
RO	0.31 + 5.9 × 10^−4^	0.30 + 5.9 × 10^−4^
RR	0.14 + 7.1 × 10^−4^	0.10 + 8.5 × 10^−4^
SMD	0.25 + 2.1 × 10^−3^	0.22 + 1.7 × 10^−3^

**Figure 1:**
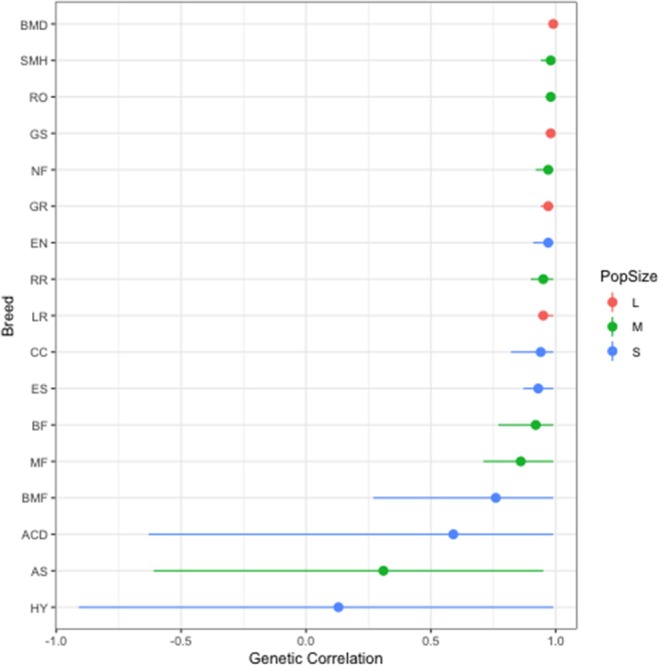
Genetic Correlation between left and right elbow by breed. The colors reflect the population size category based upon the number of dogs of a given breed evaluated: large (L), medium (M), or small (S). Values are presented as mean (dot) and the 95% confidence interval (line).

There was no statistically significant correlation between population size and heritability value. The genetic correlation between left and right elbow dysplasia varied based on breed, but was close to 1 in the majority of breeds examined. The relationship in genetic correlation between right and left elbows for each breed is displayed in Figure 1. This indicates that the heritability estimates for the left and the right elbow were not significantly different. The range was from 0.13 in hybrid dogs to 0.99 in Bernese Mountain Dogs.

**Figure 2:**
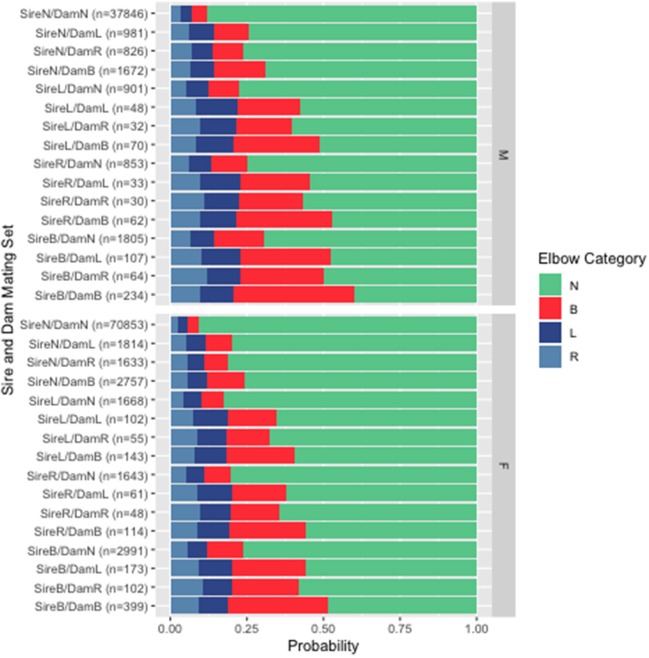
Probability that male (M) and female (F) progeny will have normal (N), left (L), right (R), or bilateral (B) elbow dysplasia as a function of elbow status of sire and dam indicated by the breeding combinations on the left. *n*, number of breeding combinations in each category.

Displayed in Figure 2 are the average probabilities of progeny to have either normal or dysplastic elbows for all possible sire and dam combinations, across the four possible offspring elbow phenotypes. These values were computed from the complete database, across all breeds, to visualize the possibility that sires and dams may have an unequal impact on progeny phenotypes.

**Figure 3:**
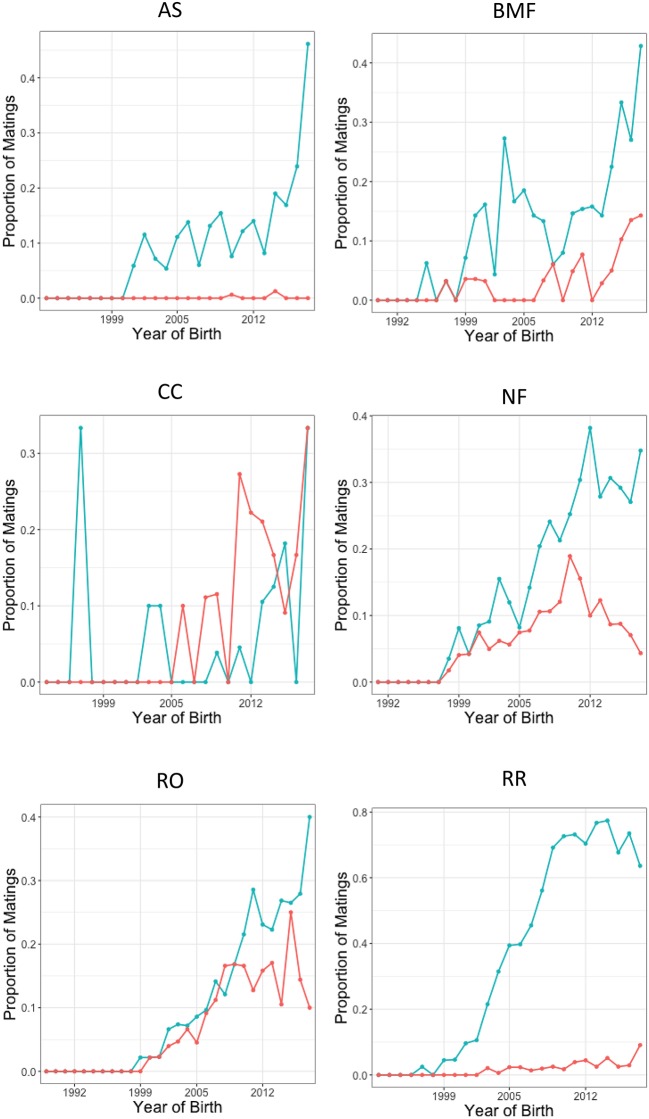
Mating proportions of different breeds (AS, BMF, CC, NF, RO, and RR) over time, recorded at the time of offspring birth. The blue line represents recorded matings of normal-normal dogs. The orange line represents recorded matings of normal-affected dogs. Note, the axes' scales differ.

Interestingly, there were also certain breeds for which there has been a continued high proportion of matings that included dysplastic dogs over the years. Figure 3, including only dogs with known elbow phenotypes, represents the trend over time of breeders' willingness to exclude dogs with elbow dysplasia from breeding programs in different breeds over time. The Chow Chow, for example, recorded an average of only 31.7% of the known mating pairs as involving two normal dogs throughout the years. That percentage further decreased when dogs of unknown or untested elbow status were included in the analyses. Only 5.3% of all total recorded breedings for the Chow Chow breed were between two normal dogs. A total of 45.5% of recorded breedings for Chow Chows were between two dogs with unknown elbow status. While the Chow Chows consisted of a small population within this study, similar findings were noted within the Rottweiler breed. Of those matings with known sire and dam elbow grades for Rottweilers, only 51.4% were between two normal dogs. When including dogs with unknown elbow phenotypes, recorded matings between two normal Rottweilers decreased to only 14.4% while 39.9% of all breedings were between two dogs with an unknown elbow status, and 3.1% of all breedings were between two dysplastic dogs.

In contrast, the Australian Shepherd breed has consistently retained only a low proportion of matings over the years involving dysplastic dogs, with no known breedings to any dogs with elbow dysplasia recorded in the most recent years. Rhodesian Ridgebacks have similar statistics, with 96% of the known, recorded breedings being between two normal dogs. If mating pairs with unknown elbow status were included in the calculations, 47% were determined to be between two normal dogs and 22% between two dogs of unknown status. This is a considerably higher proportion of normal-to-normal matings than what is observed in the breeds such as the Chow Chow or Rottweiler. Hybrid dogs were excluded from this analysis due to a low percentage of recorded parent data.

**Figure 4:**
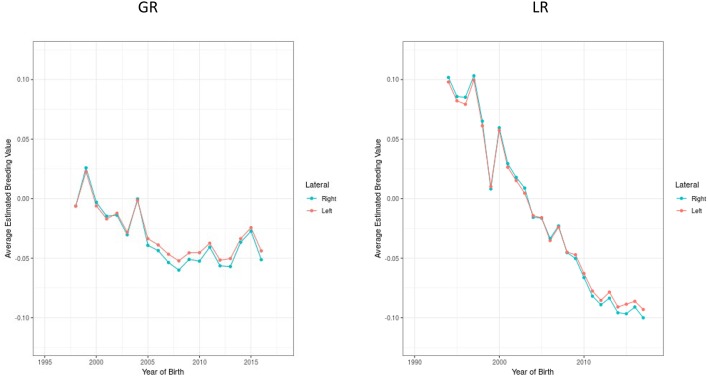
Estimated breeding values standardized for the genetic standard deviation, over time for elbow dysplasia in Golden and Labrador Retrievers.

The prevalence of disease seen in the offspring of the different combinations of matings was also calculated and is presented in Table 3. While this is a less accurate method of evaluating the inheritance pattern of elbow dysplasia than by using heritability values, it does confirm and reflect the calculated heritabilities and the risks associated with breeding affected dogs. In Rottweilers, for example, the prevalence of disease seen when breeding two normal parents was 25.2%. This increased dramatically, to 41.6%, when one parent was affected and even more so when both parents were affected (56.1%). In Labrador Retrievers, normal parents were observed to produce affected offspring 8.3% of the time. The proportion of affected offspring increased to 16.1% when one parent was also dysplastic, and increased further to 30% when both parents were dysplastic. This is repeated in the majority of the breeds evaluated, with the exception of those whose affected population was low. Bullmastiffs, for example, only had one reported pairing of two dysplastic parents, and the single offspring screened for elbow dysplasia was reported as normal. Several breeds, such as the Australian Cattle Dogs, Australian Shepherds, and Bouvier de Flanders had no reported pairings of two dysplastic dogs, and therefore the prevalence of elbow dysplasia in offspring of these pairings were falsely reported as 0%. For Golden and Labrador Retrievers, the average estimated breeding values associated with elbow dysplasia exhibited a negative trend (Figure 4).providing evidence that these breed populations are improving elbow conformation, albeit slowly when compared to changes seen for hips in these same breeds (7).

**Table 3:** Prevalence, by breed, of elbow dysplasia in offspring based on parent phenotype.

**Breed**	**Prevalence of elbow dysplasia in the offspring, given parent combinations of:**					
	**Normal × normal**	**Normal × affected**	**Affected × affected**	**Normal × unknown**	**Affected × unknown**	**Unknown × unknown**
ACD	0.071	0.222	–	0.078	0.375	0.072
AS	0.026	0.375	–	0.030	0.071	0.024
BF	0.082	0.171	–	0.090	0.077	0.089
BMD	0.184	0.304	0.389	0.230	0.329	0.246
BMF	0.179	0.143	–	0.140	0.179	0.134
CC	0.174	0.415	1.000	0.377	0.571	0.527
ES	0.111	0.289	0.778	0.112	0.329	0.140
EN	0.113	0.396	0.667	0.128	0.152	0.161
GR	0.072	0.159	0.400	0.083	0.196	0.095
GS	0.127	0.231	0.429	0.158	0.226	0.171
LR	0.083	0.161	0.30	0.085	0.152	0.097
MF	0.108	0.166	0.500	0.119	0.228	0.140
NF	0.198	0.264	0.484	0.189	0.324	0.206
RO	0.252	0.416	0.561	0.288	0.419	0.361
RR	0.050	0.075	–	0.042	0.108	0.055
SMD	0.057	0.178	0.533	0.115	0.206	0.097

## Discussion

Though there have been suggestions that there may be differences, the heritability estimates of left vs. right-sided elbow dysplasia were comparable as were the impacts of the sire or the dam on the elbow scores of the offspring. There was also no relationship between population size and heritability observed, though the heritability values varied significantly based on breed.

This study yielded either similar or slightly lower heritability estimates than have previously been reported for elbow dysplasia (9, 13). This is likely due to the heritability values being divided into left-sided and right-sided heritability rather than indicating bilateral disease or generally being categorized into elbow dysplasia. However, it is also possible that these values may have decreased, and may continue to decrease, as more breeding dogs are being health tested, and as selection pressure is applied in the majority of breed.

Some breeds, as demonstrated in Tables 2, 3, have a much greater estimated heritability and prevalence of elbow dysplasia than others. The Chow Chow and Rottweiler, for example, have a much greater risk of producing affected puppies from normal parents than does the Australian Shepherd. This may be due to the overall genetics or conformation of the breed. However, it is also possible that this reflects the diligence of the breeders themselves. It is interesting to note that those breeds with higher heritability estimates (BMD, CC, ES, GS, NF, RO, and SMD) are the same breeds that have a continued higher prevalence of elbow dysplasia. These are also the breeds that have higher proportions of affected dogs included in breeding pairs, or those that have an increased number of pairings between dogs with unknown sire and/or dam elbow status. Chow Chows, for example, have a high proportion of breeding pairs that include a dog with elbow dysplasia. It is unclear if this trend is due to the poor compliance or culture within the Chow breeders, or if the proportion of affected dogs is so high among within this breed that eliminating all of these dogs from the breeding pool would be detrimental to the sustainability of the breed itself. Given the higher heritability values in these breeds, it is reasonable to conclude that the prevalence of elbow dysplasia could be decreased with more widespread screening of the parents and more careful selection of breeding normal dogs. Considering the number of affected dogs within these populations, strictly limiting breeding pairs to only unaffected dogs would severely limiting the genetic pool, which is undesirable. However, if breeders continue to breed affected dogs, even those with grade I elbow dysplasia, then the prevalence of elbow dysplasia within the breed is unlikely to decrease substantively and elbow dysplasia will continue to be an issue within these breeds. Additionally, for those breeds with a higher proportion of normal to normal matings, it is unclear if this was due to the overall low incidence of elbow dysplasia in these breeds, or if the low incidence and lower heritability values of elbow dysplasia are because of the continued compliance in breeding dogs with normal elbows. Rhodesian Ridgebacks similarly have had a historically low proportion of affected dogs being bred, and have a relatively low heritability value for elbow dysplasia.

Improvements in the incidence of both hip and elbow dysplasia over time have been noted, despite the screening process and compliance to selective breeding being entirely voluntary (9). The BVA has also reported similar findings within the population of dogs submitted for evaluation. The overall percentage of dogs in the UK with normal elbows has increased steadily from 70.0% in 1999 to 84.4% in 2016. The number of overall submissions has also increased dramatically from 583 in 1999 to 4,176 dogs in 2016 (21). The New Zealand Veterinary Association (NZVA) reported small but favorable genetic trends in reducing elbow dysplasia in German Shepherds, Rottweilers, and Golden Retrievers between 1992 and 2013 (13) as was seen in a Swedish population (22). Unfortunately, eliminating dogs with grade II or grade III elbow dysplasia from the breeding pool only excludes ~4–8% of dogs, which does not exert a high selection pressure to rapidly improve elbow conformation (7, 15). This is in contrast to hip dysplasia statistics, where approximately 18% of dogs are eliminated from breeding programs due to inadequate hip formation (7). The improvement in elbow dysplasia has all been achieved by selecting phenotypically normal dogs for use in breeding. However, elbow dysplasia is a complex, multi-factorial disease, and even with two normal parents there is no guarantee for disease-free puppies. To overcome this limitation, estimated breeding values (EBV), based upon familial expression of the disease, can be used to improve the predictability that a given dog may pass on a disease/condition to its offspring. The EBV of a dog factors in the quality of both the individual dog's parents, their relatives and offspring produced, which is considered a more accurate representation of the dog's genetic quality than an individual record (23). EBVs may increase the rate of improvement in elbow or hip dysplasia within the population. However, EBVs are just starting to become more available in many countries through kennel clubs and are not yet commonly used among the majority of breeders in the USA (24). For the two breeds in this report with sufficient numbers of elbow evaluations to assess the genetic propensity contributing to elbow dysplasia, EBVs decreased over time and the genetic progress for Labrador retrievers was of a similar magnitude seen in that breed assessed in the United Kingdom (7). Implementation of more widespread diagnostics and/or EBVs could substantially reduce the prevalence of elbow dysplasia. This study confirmed the importance of ensuring both the sire and the dam have appropriate elbow clearances prior to breeding.

In this study, the calculations performed did not subdivide the dysplastic dogs into their respective grade of elbow dysplasia or primary disease process, largely out of concern for losing analytical power because of low numbers of affected dogs in each group. Therefore, the heritability values calculated were an average of all abnormal grades. It has previously been shown that the percentage of affected offspring increase with the severity of disease in the parent (25), so it is suspected that there may be additional genetic factors that influence inheritance or expression of these traits in the offspring of more severely affected dogs. This also may have precluded the detection of any maternal or paternal effects, as a maternal effect has been suggested in the inheritance of fragmented medial coronoid disease. The lack of a distinct maternal effect on the risk of the progeny inheriting elbow dysplasia within this study is in contrast to a previous study (12). No overall maternal or paternal effects were observed in this study when grouping all abnormalities into elbow dysplasia. Any further analyses on individual subcategories would require a larger population of affected dogs for more accurate heritability values and further study of parental effects.

The low genetic correlation value seen between the left and right side estimates in the Hybrids (0.13), Australian Shepherds (0.31), and Australian Cattle Dogs (0.59) was most likely due to the smaller sample size and the limited number of affected dogs in these breeds. For example, out of 3,346 Australian Shepherds, only 53 elbows were graded as unilaterally dysplastic. Additional research with a larger affected population size would be necessary to determine if the findings were reflective of true differences within these breeds.

There were limitations to the study in that it was a retrospective study, and planned breedings between different elbow phenotypes with subsequent follow-up of all progeny were not done. As a retrospective study, there was no control for factors in image acquisition (such as increasing age or positioning) or for environmental factors such as the dog's activity or nutrition. Utilizing a large number of dogs with over a number of years and generations was done to counteract that those limitations. Also, it is likely that data used in the study were biased to a degree, as some owners will not submit radiographs if it is obvious that the dog is dysplastic. However, this study utilized the abnormal elbow grades that were not made available to the public, and therefore the bias was minimized. Furthermore, a substantial number of dogs were investigated in this study, and the trends remain consistent with those of previous studies.

Additionally, the diagnosis of elbow dysplasia is based upon the presence of one of several conditions (FCP, UAP, OCD, joint incongruity). These conditions reflect different developmental anomalies, but are all classified more generally as elbow dysplasia. The complexity of the multiple possible disease processes grouped together under the category of elbow dysplasia contributes to low heritabilities and slower genetic progress in reducing the overall incidence of elbow dysplasia. These separate disease complexes were not considered individually during this study.

The use of varying diagnostic methods to diagnose elbow dysplasia was not differentiated in this study, and the diagnosis of the final grade these dogs received was based on a minimum of a single flexed lateral radiograph of each elbow. The use of radiographs provides owners and breeders with a feasible and overall well-accepted method of phenotypic screening. However, it is widely accepted that computed tomography and arthroscopy are of higher diagnostic quality and are considered the gold standard for diagnosing elbow dysplasia (26, 27). This poses an ethical and political conflict, as computed tomography (CT) requires anesthesia and is more costly to the owners than is a radiographic exam, which may be taken awake or with varying degrees of sedation. The IEWG currently does not recognize a standardized method of obtaining CTs of the elbow to be used in the screening process, as joint congruency may still be affected by positioning and slice thickness of the CT used. Thus, in the absence of standardization, at this time the OFA, the FCI, and the BVA do not accept the use of CT to diagnose elbow dysplasia (26). With only one or two radiographic views required for screening, it is possible that some lesions may have been present but were not visible on radiographs (particularly only on a single lateral view). This would result in a small proportion of dogs rated as normal that perhaps should not have received a passing grade, and therefore are not eliminated from breeding programs. A standardized protocol for elbow CTs would be required in the future in order to use CT imaging as an alternative in health screening programs, particularly if the dog is borderline or questionable on elbow radiographs (27, 28).

In conclusion, as evidenced by the point estimates of heritability and the associated credible intervals that can be generated by the variability of these estimates, there were no substantive differences between the heritability of the left vs. right elbow, or of the sire vs. dam influence. Dogs with a particular unilaterality did not have a higher risk of passing down the same laterality to their offspring, although the risk of elbow dysplasia itself increased in the offspring when one or both parents were affected. While there have been some improvements in reducing the incidence of elbow dysplasia across many breeds since the initiation of phenotypic screening tools, the progression is significantly slower than the improvements seen in other diseases. Employment of widespread screening, judicious use of dogs in breeding programs, and the development and incorporation of EBVs may accelerate improvement. Elbow dysplasia can be a significant health concern for the affected dog and treatments are largely ineffective, which leads to control of this disease being based on selectively breeding for normal dogs. Therefore, control of the disease falls to the breeder's responsibility to have dogs tested and to make appropriate decisions on the suitability of their individual dogs for breeding in order to promote healthier generations of puppies.

## Data Availability Statement

The datasets generated for this study are available on request to the corresponding author.

## Ethics Statement

Ethical review and approval was not required for the animal study because the manuscript assessed only owner-submitted data without identifiers. No animals were directly involved with the study, only data. Written informed consent for participation was not obtained from the owners because no consent was necessary as the owners freely submitted the data to the health registry knowing that the data can be used for evaluative purposes.

## Author Contributions

Experimental design by GB, GK, and AO. Data analyses by GB, TF, and AO. Manuscript drafting by GB and AO with editing by all authors.

### Conflict of Interest

GK is the Chief Veterinary Officer of the Orthopedic Foundation for Animals. GB is a radiologist resident working at the Orthopedic Foundation for Animals. AO is on the board of directors for the Orthopedic Foundation for Animals. This relationship did not influence the interpretation or presentation of the data in the manuscript. The remaining author declares that the research was conducted in the absence of any commercial or financial relationships that could be construed as a potential conflict of interest.
